# Iron Chelation by Deferoxamine Prevents Renal Interstitial Fibrosis in Mice with Unilateral Ureteral Obstruction

**DOI:** 10.1371/journal.pone.0089355

**Published:** 2014-02-19

**Authors:** Yasumasa Ikeda, Iori Ozono, Soichiro Tajima, Mizuki Imao, Yuya Horinouchi, Yuki Izawa-Ishizawa, Yoshitaka Kihira, Licht Miyamoto, Keisuke Ishizawa, Koichiro Tsuchiya, Toshiaki Tamaki

**Affiliations:** 1 Department of Pharmacology, Institute of Health Biosciences, The University of Tokushima Graduate School, Tokushima, Japan; 2 Student Lab, The University of Tokushima Faculty of Medicine, Tokushima, Japan; 3 Department of Medical Pharmacology, Institute of Health Biosciences, The University of Tokushima Graduate School, Tokushima, Japan; Biomedical Research Foundation of the Academy of Athens, Greece

## Abstract

Renal fibrosis plays an important role in the onset and progression of chronic kidney diseases (CKD). Although several mechanisms underlying renal fibrosis and candidate drugs for its treatment have been identified, the effect of iron chelator on renal fibrosis remains unclear. In the present study, we examined the effect of an iron chelator, deferoxamine (DFO), on renal fibrosis in mice with surgically induced unilateral ureter obstruction (UUO). Mice were divided into 4 groups: UUO with vehicle, UUO with DFO, sham with vehicle, and sham with DFO. One week after surgery, augmented renal tubulointerstitial fibrosis and the expression of collagen I, III, and IV increased in mice with UUO; these changes were suppressed by DFO treatment. Similarly, UUO-induced macrophage infiltration of renal interstitial tubules was reduced in UUO mice treated with DFO. UUO-induced expression of inflammatory cytokines and extracellular matrix proteins was abrogated by DFO treatment. DFO inhibited the activation of the transforming growth factor-β1 (TGF-β1)-Smad3 pathway in UUO mice. UUO-induced NADPH oxidase activity and p22^phox^ expression were attenuated by DFO. In the kidneys of UUO mice, divalent metal transporter 1, ferroportin, and ferritin expression was higher and transferrin receptor expression was lower than in sham-operated mice. Increased renal iron content was observed in UUO mice, which was reduced by DFO treatment. These results suggest that iron reduction by DFO prevents renal tubulointerstitial fibrosis by regulating TGF-β-Smad signaling, oxidative stress, and inflammatory responses.

## Introduction

The incidence of chronic kidney disease (CKD) has increased worldwide. CKD worsens morbidity and mortality in the general population [1], [2]. Additionally, the progression of CKD results in end-stage renal failure, which requires treatment by hemodialysis. Several factors are involved in the onset and progression of CKD. The process of renal tubulointerstitial fibrosis is characterized by extracellular matrix deposition, interstitial myofibroblast proliferation, and the infiltration of inflammatory mononuclear cells, which are thought to play an important role in the pathogenesis of CKD [3]. Therefore, preventing renal interstitial fibrosis is important for inhibiting the progression of CKD.

Iron is an elementary trace metal that is essential for nearly all organisms. Excess iron, however, causes oxidative stress through the production of hydroxyl radicals via Fenton/Haber–Weiss catalytic reactions [4], which in turn cause tissue damage. Therefore, the level of intracellular iron is regulated by iron transporters and iron-binding proteins, and iron is stored in metalloproteins, heme complexes, oxygen carrier proteins, and other complexes under normal physiological conditions [5].

Patients with iron overload diseases, such as hereditary hemochromatosis or thalassemia, suffer complications such as cardiomyopathy, liver cirrhosis, and diabetes mellitus following ectopic iron accumulation in the heart, liver, and pancreas, respectively [6]. Recent studies have shown that iron also contributes to pathology in patients with non-iron overload disorders such as hepatitis C [7], [8] and Alzheimer’s disease [9], [10], and that iron reduction therapy can ameliorate these disorders [7], [8], [11], [12]. Iron reduction also has preventive effects in other diseases, including cardiovascular remodeling [13]–[15], obesity [16], and diabetes [17], [18]. Thus, iron is involved in the pathogenesis of hereditary iron overload diseases as well as in various non-iron overload diseases.

In studies of the relationship between kidney disease and iron, angiotensin II (AngII) administration increased renal iron deposition and altered the expression of renal iron transporters in rats [19], [20]. Dietary iron restriction can prevent renal injury by inhibiting mineralocorticoid receptor signaling in rats with CKD modeled with 5/6 nephrectomy [21], [22]. Additionally, we previously demonstrated the beneficial effects of a low-iron diet on the progression of diabetic nephropathy [23]. These findings strongly suggest that iron is involved in renal damage and that iron reduction ameliorates kidney injury. However, the effect of iron reduction on renal tubulointerstitial fibrosis remains unclear.

Deferoxamine (DFO), a bacteria-derived siderophore, chelates iron by binding iron in the blood and excreting it as a DFO-iron complex in urine or stool [24]. The preventive effects of DFO on the progression of obesity [16] and AngII-induced cardiovascular fibrosis have been demonstrated [13], [14]. On the other hand, DFO upregulates hypoxia-inducible factor-1α (HIF-1α) activity [25], and HIF-1α is an aggravating factor in tubulointerstitial renal injury [26]. DFO also increases collagen I and tissue inhibitor metalloproteinase 1 mRNA levels in vitro [27]. Furthermore, iron chelation abolishes IL-10-mediated protection against renal injury [28]. Thus, the effect of DFO on kidney disease remains controversial.

In the present study, we demonstrated that iron chelation using DFO alleviated renal tubulointerstitial fibrosis in mice with unilateral ureteral obstruction (UUO). Furthermore, DFO suppressed UUO-induced renal oxidative stress, inflammation, and transforming growth factor-β1 (TGF-β1)-Smad signaling. Our results suggest that iron chelation could be a new therapeutic approach for treating renal fibrosis.

## Materials and Methods

### Chemicals and Reagents

DFO was purchased from Calbiochem (San Diego, CA, USA). The following commercially available antibodies were used in this study: anti-fibronectin (SC-6952), anti-p22^phox^ (SC-20781), anti-TGF-β1 (SC-146), anti-IL (interleukin)-1β (SC-7884), anti-ferritin heavy chain (FTH) (SC-14416), anti-ferritin light chain (FTL) (SC-14422), anti-collagen IA (SC-25974), anti-collagen IIIA (SC-8780R), and anti-NRAMP2 (divalent metal transporter-1; DMT1) (SC-30120) antibodies (Santa Cruz Biotechnology, Santa Cruz, CA, USA); anti-α-smooth muscle actin (αSMA) antibody (A2547) (Sigma-Aldrich, St. Louis, MO, USA); anti-rat F4/80 antibody (MCA497GA) (AbD Serotec, Oxford, UK); anti-phospho Smad3 (9520), anti-total Smad3 (9523), and anti-monocyte chemoattractant protein-1 (MCP-1) (2029) antibodies (Cell Signaling Technology, Danvers, MA, USA); anti-transferrin receptor 1 (TfR) antibody (13–6800) (Life Technologies, Carlsbad, CA); anti-ferroportin (FPN) antibody (MTP11-A) (Alpha Diagnostics, San Antonio, TX, USA); anti-NADPH oxidase 4 (NOX4) (ab109225) and anti-collagen IV (ab6586) antibodies (Abcam, Cambridge, MA); and anti-tubulin antibody (CP06) (Calbiochem, San Diego, CA, USA) as a loading control.

### Experimental Animals and Treatment

All animal experimental procedures were performed in accordance with the guidelines of the Animal Research Committee of the University of Tokushima Graduate School, and the protocol was approved by the Tokushima University Institutional Review Board for animal protection (Permit Number: 12022). Male C57BL/6J mice were purchased from CLEA Japan Inc. (Tokyo, Japan). Mice were maintained in a room under conventional conditions with a regular 12-h light/dark cycle, and were given free access to food (Type NMF; 10 mg Fe/100 g food; Oriental Yeast, Tokyo, Japan) during the study. At 8–12 weeks of age, the mice underwent surgery to induce unilateral ureteral obstruction (UUO). UUO is a well-established experimental model of tubulointerstitial fibrosis. Briefly, under pentobarbital anesthesia, the left ureter was exposed through a lateral incision and ligated with 3-0 silk at 2 points at the proximal site. In sham mice, the left ureter was exposed but not ligated. The total operation period was approximately 10 min. After the operation, the mice were placed on a heat mat until the animals completely recovered. Sham and UUO mice were divided into 2 groups and intraperitoneally injected with vehicle (VEH) or DFO (100 mg·kg^−1^·day^−1^) for 7 days immediately after operation (sham+VEH, *N* = 18; sham+DFO, *N* = 14; UUO+VEH, *N* = 15; UUO+DFO, *N* = 15).

### Peripheral Blood Analysis

Complete blood counts were performed by Shikoku Chuken Co., Ltd. (Kagawa, Japan).

### Histological Analysis

Seven days after surgery, mice were sacrificed by intraperitoneal injection of excess pentobarbital, and the kidneys were excised after normal saline perfusion. A portion of each kidney was fixed overnight in 4% paraformaldehyde at 4°C and embedded in paraffin. Samples were cut into 3 µm sections and stained with Masson’s trichrome to evaluate renal interstitial fibrosis. Fourteen fields were randomly selected in 5 different sections of the renal cortex area. The fibrotic area was quantified by manually tracing the blue-stained area; the total scanned area, excluding the tubular lumen, glomeruli, and vessels, was also quantified using ImageJ 1.38x software (National Institutes of Health, Bethesda, MD). The fibrotic fraction volume ratio was expressed as the interstitial area relative to the total area.

### Measurement of Tissue Iron Concentration

The uncentrifuged crude lysate of the whole kidney was used for iron measurement. Iron concentrations were measured using a Metallo Assay kit (AKJ Global Technology, Chiba, Japan) as previously described [23], [29]. The renal iron concentration was corrected by wet weight and expressed as microgram per gram of tissue.

### Western Blotting

Protein expression was evaluated with western blotting according to methods previously described in detail [16]. In brief, tissues were homogenized in lysis buffer with protease and phosphatase inhibitors, and the proteins were extracted. The samples were boiled for 5 min in Laemmli sample buffer, separated using SDS-PAGE, and transferred to a polyvinylidene fluoride membrane. A chemiluminescence reagent was used to detect immunoreactive bands. ImageJ 1.38x software was used for semi-quantitative densitometric analysis of the bands. The density of each band was normalized to that of the tubulin band. Non-denatured and non-reduced samples were used for the detection for collagen IA and IIIA proteins. The intensities of collagen IA and IIIA bands were normalized to the intensity of a membrane protein band (MemCode Reversible Protein Stain kit; Thermo Fisher Scientific Inc., Waltham, MA). The antibodies were used at the following dilutions: anti-collagen IA (1∶100), anti-collagen IIIA (1∶250), anti-collagen IV (1∶500), anti-MCP-1 (1∶100), anti-IL-1β (1∶250), anti-αSMA (1∶1500), anti-fibronectin (1∶500), anti-p22^phox^ (1∶250), anti-NOX4 (1∶500), TGF-β1 (1∶250), anti-phospho Smad3 (1∶250), anti-total Smad3 (1∶1000), anti-TfR (1∶1000), anti-DMT1 (1∶250), anti-FPN (1∶500), anti-FTH (1∶250), anti-FTL (1∶250), and anti-tubulin (1∶1000).

### Immunohistochemistry

Paraffin-embedded kidney samples were sectioned, deparaffinized, and processed with antigen retrieval in 10 mM citrate buffer at 95°C for 10 min or 0.05% trypsin in phosphate-buffered saline at 37°C for 20 min. The sections were then incubated with primary antibody at 4°C overnight. Antibody distribution was visualized using a streptavidin-biotin complex assay and a DAB substrate kit (LSAB+ Kit Universal; Dako Japan, Tokyo, Japan). To evaluate renal macrophage infiltration, 10 fields were randomly selected in the renal cortex area, and the macrophage-positive area was expressed as a percentage of the whole area, excluding the tubular lumen, glomeruli, and vessels, using ImageJ 1.38x software analysis. The following antibodies were used: anti-F4/80 (1∶500), anti-αSMA (1∶200), anti-fibronectin (1∶100), anti-p22^phox^ (1∶100), anti-TfR (1∶200), anti-DMT1 (1∶100), anti-FPN (1∶100), anti-FTH (1∶100), and anti-FTL (1∶100).

### Measurement of NADPH Oxidase Activity

NADPH oxidase activity was measured as previously described [16], [23]. Briefly, the kidney sample was weighed, immediately homogenized in lysis buffer, and then sonicated for 3 s. NADPH substrate (100 µM) was added to a renal suspension with lucigenin (10 µM). Luminescence was measured every second for 60 min in a plate reader (SpectraMax Paradigm FilterMaxF3; Molecular Devices Japan, Tokyo, Japan). NADPH activity was expressed as relative luminescence units normalized to the protein concentration.

### Statistical Analysis

Data are shown as the mean ± standard error of the mean (SEM). For comparisons among 4 groups, the significance of each difference was evaluated by post-hoc test using the Tukey-Kramer method. *P-*values <0.05 were considered statistically significant.

## Results

### Characteristics of the mice: Body Weight, Kidney Weight, Hematological Parameters, and Iron Concentration

Mice from the 4 groups were examined after treatment with DFO or vehicle. As shown in Table 1, there were no differences in body weight, hemoglobin levels, and hematocrit levels among the 4 groups. The right kidney weights of UUO+VEH and UUO+DFO mice were significantly greater than those of sham+VEH and sham+DFO mice. On the other hand, the left kidney weights of UUO+VEH mice were lighter than those of sham+VEH or sham+DFO mice. The reduced left renal weight in UUO mice was restored by treatment with DFO. The renal iron concentration was significantly higher in UUO mice treated with vehicle, and treatment with DFO reduced the renal iron content in UUO mice.

**Table 1:** Body weight, kidney weight, hemoglobin content, and kidney iron content in mice, 7 days after surgery.

	Sham with vehicle (*n* = 18)	Sham with DFO (*n* = 14)	UUO with vehicle (*n* = 15)	UUO with DFO (*n* = 15)
Body weight (BW) (g)	26.8±0.8	25.9±0.3	26.6±0.5	26.6±0.8
Right kidney weight (RKW) (mg)	136±4##	137±2##	155±3** ††	158±3** ††
RKW to BW ratio	5.2±0.1##	5.3±0.1##	6.1±0.1** ††	6.2±0.2** ††
Left kidney weight (LKW) (mg)	135±5##	131±3#	116±1** †	134±5#
LKW to BW ratio	5.0±0.1#	5.0±0.1#	4.6±0.1* †	5.2±0.2##
Hemoglobin (g/dL)	13.8±0.3	12.8±0.1	13.1±0.3	13.6±0.2
Hematocrit (%)	42.3±0.8	39.7±0.4	40.3±1.0	41.3±0.7
Left kidney iron (µg/g kidney tissue)	5.9±0.6#	4.8±0.7##	9.0±1.2* ††	4.9±0.6##

Data are the mean ± SEM, *n* = 14–18, as indicated.

*P<0.05,

**P<0.01 vs. sham with vehicle;

† *P*<0.05,

†† *P*<0.01 vs. sham with DFO;

# *P*<0.05,

## *P*<0.01 vs. UUO with vehicle.

### Effect of DFO Administration on UUO-induced Renal Interstitial Fibrosis

**Figure 1:**
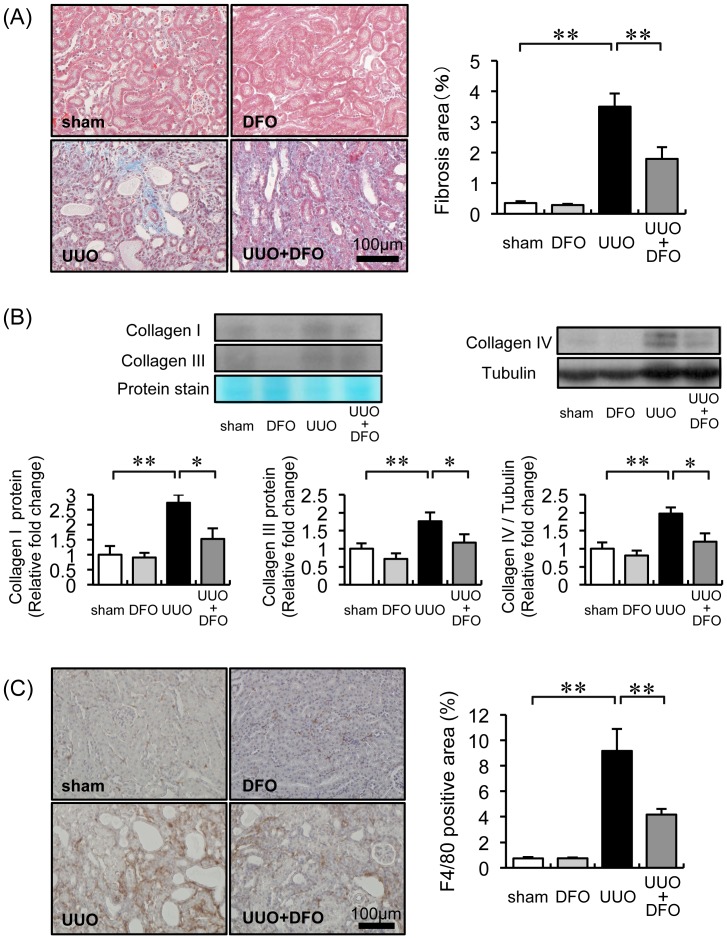
Effect of DFO on UUO-induced renal interstitial fibrosis, collagen expression, and macrophage infiltration. (A) Representative histological findings in tissue stained with Masson’s trichrome 7 days after surgery. (B) Quantitative analysis of renal interstitial fibrosis at day 7. Sham+VEH (white bar), sham+DFO (light gray bar), UUO+VEH (black bar), and UUO+DFO (gray bar). Results are expressed as the mean ± SEM. *P<0.05, **P<0.01. *n = *12 per group. (C) Analysis of the renal expression of collagen IA, collagen IIIA, and collagen IV. Upper panels: representative immunoblotting for collagen IA, collagen IIIA, and collagen IV. Lower panels: quantitative analysis of collagen IA, collagen IIIA, and collagen IV expression normalized to protein staining or tubulin. Results are expressed as the mean ± SEM. *P<0.05, **P<0.01. *n = *12 per group. (D) Representative immunohistochemistry with F4/80 antibody 7 days after surgery. (E) Quantitative analysis of the F4/80-positive area within the interstitial area. Results are expressed as the mean ± SEM. *P<0.05, **P<0.01. *n = *12 in each group.

To examine renal fibrosis induced by UUO, we performed Masson’s trichrome staining. Seven days after surgery, the progression of renal interstitial fibrosis was greater in UUO+VEH mice (3.50±0.43%) than in sham+VEH and sham+DFO mice (0.34±0.06% and 0.28±0.03%, respectively; sham vs. UUO+VEH, *P*<0.01). Fibrotic progression in UUO mice was mitigated by DFO treatment (1.79±0.39%; *P*<0.01 vs. UUO+VEH) (Fig. 1A). Consistent with these morphological changes, the expression of collagen I, III, and IV was approximately 2.0-fold higher in the kidneys of UUO mice treated with vehicle, but this increase was attenuated to the expression level in sham mice when UUO mice were treated with DFO (UUO+VEH vs. UUO+DFO, *P*<0.05) (Fig. 1B).

### Renal Interstitial Macrophage Infiltration and Inflammatory Cytokines

**Figure 2:**
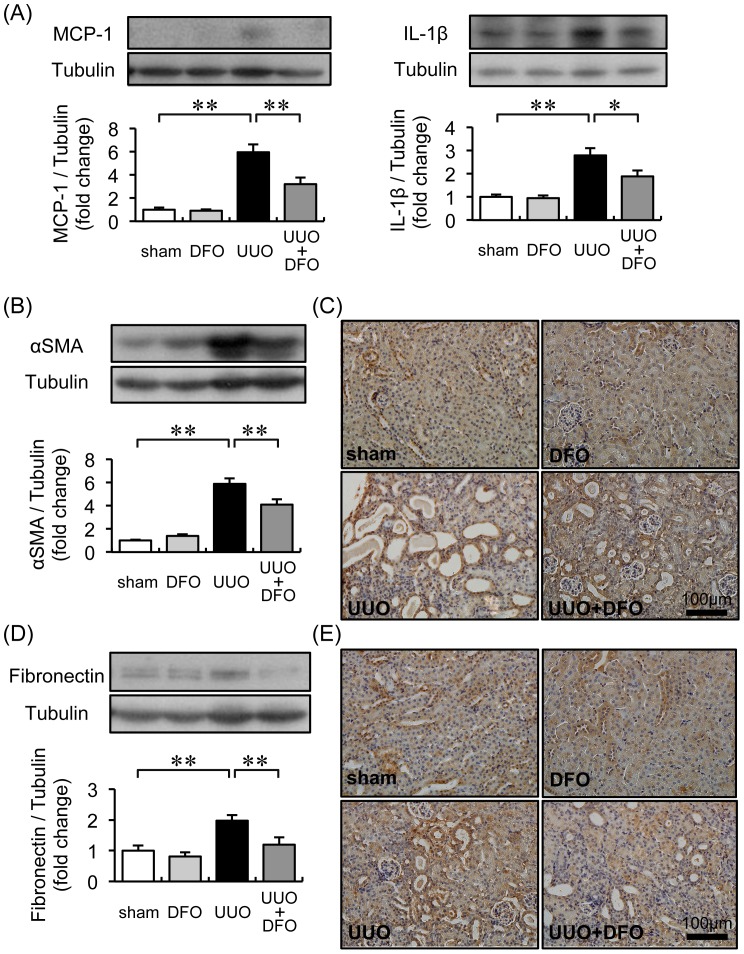
Effect of iron chelation on protein expression related to inflammation and the extracellular matrix. (A) Analysis of the renal expression of MCP-1 and IL-1β. Upper panels: representative immunoblotting for MCP-1 and IL-1β. Lower panels: quantitative analysis of MCP-1 and IL-1β expression normalized to tubulin. Results are expressed as the mean ± SEM. *P<0.05, **P<0.01. *n = *12 per group. (B) Quantitative analysis of αSMA expression. Results are expressed as the mean ± SEM. *P<0.05, **P<0.01. *n = *12 per group. (C) Representative immunochemistry results for αSMA expression in kidney sections. (D) Quantitative analysis of fibronectin expression normalized to tubulin. Results are expressed as the mean ± SEM. *P<0.05, **P<0.01. *n = *12 per group. (e) Representative immunochemistry results for fibronectin expression in kidney sections.

To investigate the effect of iron chelation on inflammation in UUO-induced renal interstitial fibrosis, we analyzed macrophage infiltration by immunohistochemistry and cytokine expression by western blot. An increase in the increment area of interstitial macrophage infiltration was observed in UUO+VEH mice (9.17±1.73%; *P*<0.01 vs. sham+VEH, 0.74±0.11%; sham+DFO, 0.75±0.05%), but was significantly reduced in UUO+DFO mice (4.17±0.46%; *P*<0.01 vs. UUO+VEH) (Fig. 1C). Consistent with attenuated macrophage infiltration, the 6-fold increase in MCP-1 and 3-fold increase in IL-1β protein expression in the kidneys of UUO mice were reduced by half when UUO mice were treated with DFO (*P*<0.01 and *P*<0.05, respectively, vs. UUO+VEH) (Fig. 2A).

### Effect of DFO on Myofibroblasts and Extracellular Matrix Expression

Consistent with the histological findings, the expression of αSMA and fibronectin was increased 6.0-fold and 2.0-fold, respectively, in the kidneys of UUO+VEH mice, compared to expression in sham+VEH mice and sham+DFO mice (*P*<0.01 vs. UUO+VEH). DFO treatment suppressed the UUO-induced interstitial accumulation of myofibroblasts and of the extracellular matrix protein (*P*<0.01, UUO+DFO vs. UUO+VEH; Fig. 2B).

### Iron Chelation Reduced UUO-induced Renal Oxidative Stress

**Figure 3:**
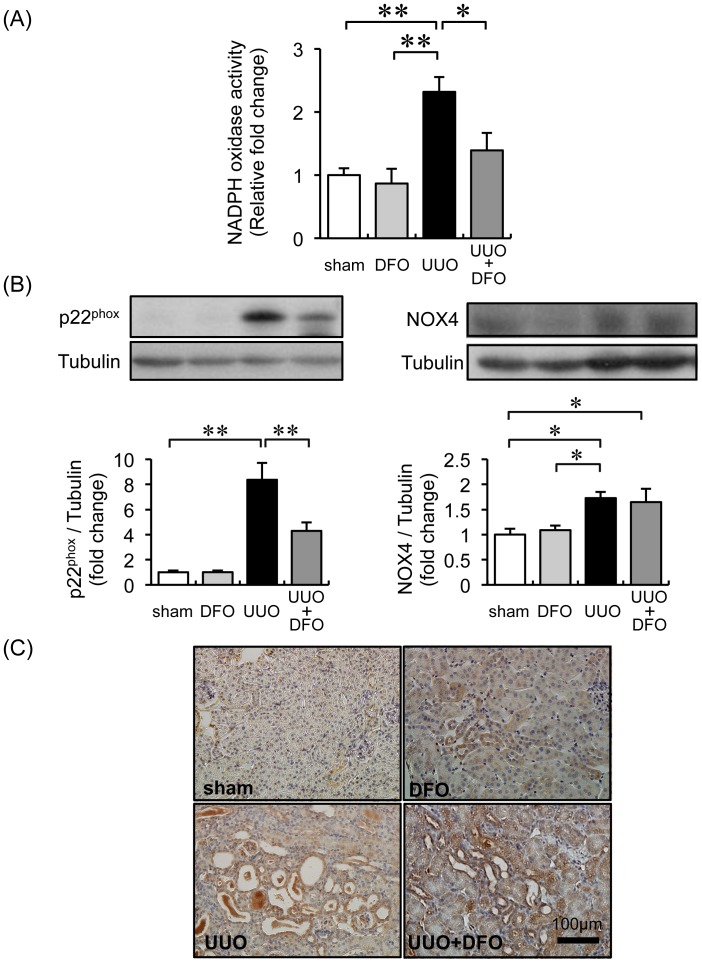
Effect of iron reduction on oxidative stress in the kidneys of mice with UUO. (A) Renal NADPH activity. Data are expressed as the mean ± SEM. *P<0.05. *n* = 4–6 per group. (B) Immunoblot analysis for p22^phox^ and NOX4 protein expression. Upper panels: representative immunoblotting for p22^phox^ and NOX4 expression. Lower panels: quantitative densitometry analysis of p22^phox^ and NOX4 normalized to tubulin. Values are expressed as the mean ± SEM. *P<0.05, **P<0.01. *n* = 12 per group. (C) Representative immunohistochemical staining of p22^phox^ expression.

Iron chelation decreases oxidative stress, and the progression of renal fibrosis is associated with oxidative stress. Therefore, we examined the effect of DFO treatment on UUO-induced oxidative stress. As shown in Fig. 3A, the renal NADPH oxidase activity of UUO mice was increased 2.3-fold (*P*<0.01 vs. sham+VEH or sham+DFO); this augmentation was abrogated by DFO treatment (*P*<0.05 vs. UUO+VEH). In immunohistochemical analysis, expression of p22^phox^ was significantly increased in the renal tubules of UUO mice treated with vehicle or DFO; this increase was mitigated by DFO treatment. Similarly, the expression of p22^phox^ in the kidney was 8.4-fold higher in UUO+VEH mice (*P*<0.01 vs. sham+VEH or sham+DFO); the expression of p22^phox^ in UUO mice decreased by half when they were treated with DFO (*P*<0.01 vs. UUO+VEH) (Fig. 3B and C). Finally, NOX4 expression in the kidney was 1.5-fold higher in UUO mice (*P*<0.01 vs. sham+VEH and sham+DFO). There was no difference in renal NOX4 expression in UUO+VEH and UUO+DFO mice (Fig. 3B).

### Effect of Reduced Iron Content on the TGF-β-Smad Signaling Pathway

**Figure 4:**
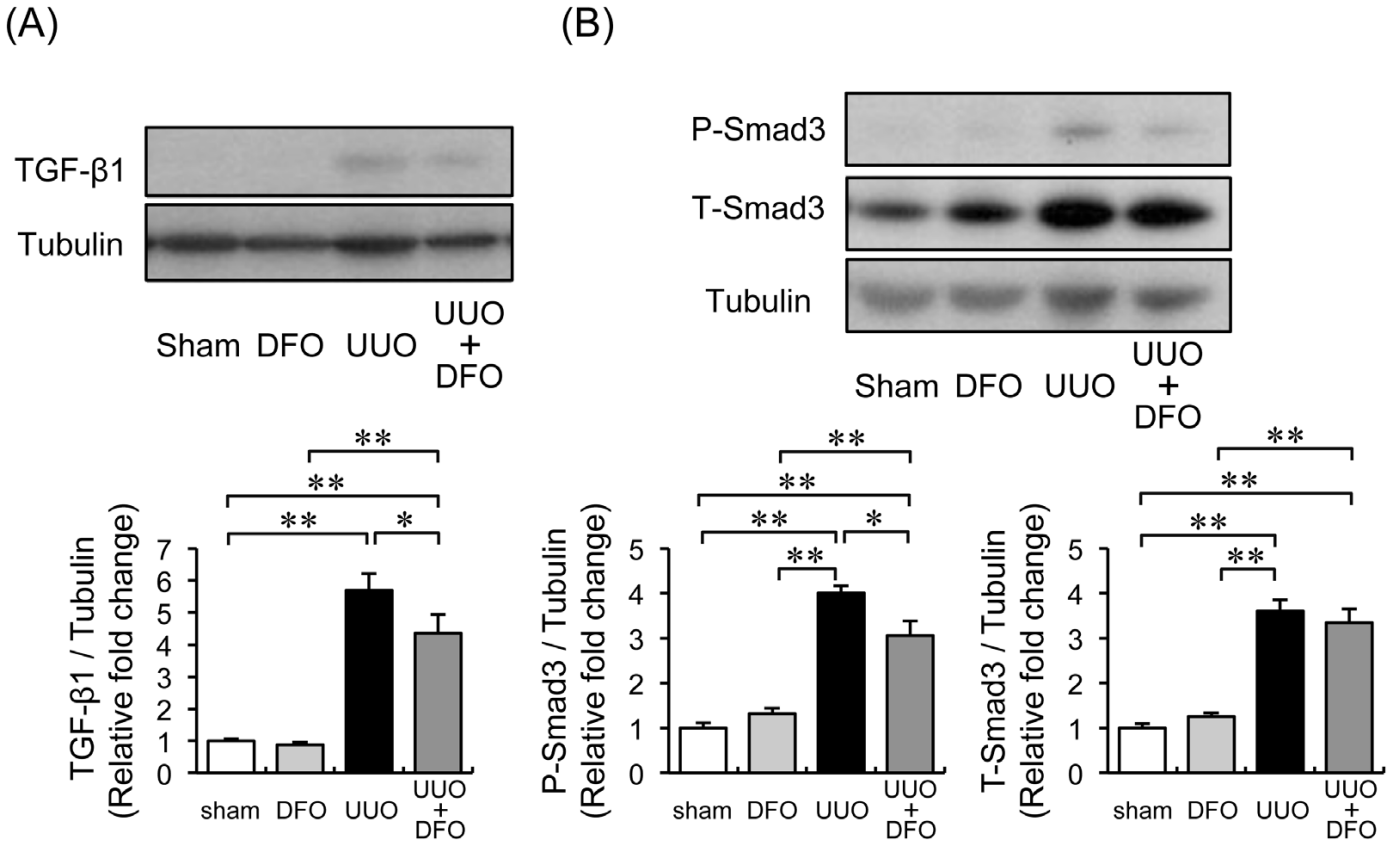
Effect of DFO on the UUO-induced acceleration of TGF-β-Smad pathway. (A) Upper panels: representative immunoblotting for TGF-β1 expression. Lower panels: quantitative evaluation of TGF-β1 protein expression at day 7. Results are expressed as the mean ± SEM. *P<0.05, **P<0.01. *n = *12 in each group. (B) Upper panel: representative western blots of phopsho-Smad3, total-Smad3, and tubulin. Lower panel: quantification of western blots. Results are expressed as the mean ± SEM. *P<0.05, **P<0.01. *n = *12 per group.

TGF-β-Smad signaling is a crucial pathway in the development of tissue fibrosis. Therefore, we examined whether the protective effect of iron reduction by DFO on UUO-induced renal fibrosis was associated with the TGF-β-Smad pathway. Renal TGF-β1 expression increased 5.7-fold in vehicle-treated UUO mice, when compared with expression in vehicle- or DFO-treated sham mice (*P*<0.01). DFO treatment reduced TGF-β1 expression to 77% of the level in UUO+VEH (Fig. 4A). The expression of phosphorylated and total Smad3 was upregulated 4.0-fold and 3.6-fold, respectively, in vehicle-treated UUO mice. DFO treatment reduced phosphorylated Smad3 levels to 76% of the level in UUO+VEH. DFO treatment did not alter total Smad3 expression in UUO mice (Fig. 4B).

### Changes in the Expression of Iron Transporters, Ferritin, and Urinary Iron Excretion in UUO-induced Fibrotic Kidney

**Figure 5:**
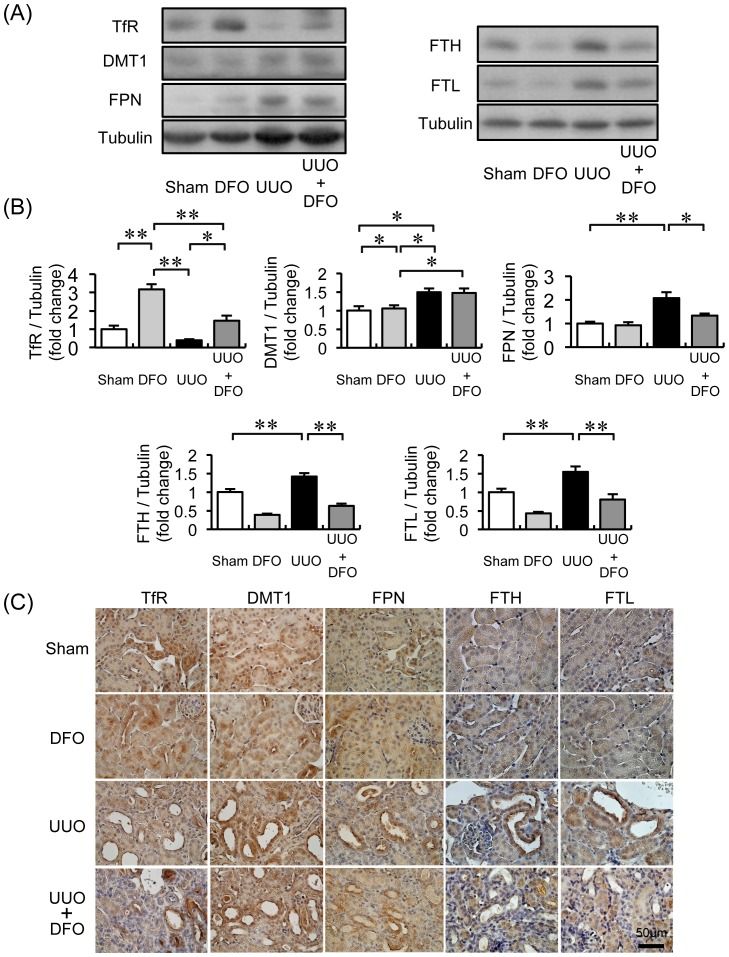
Changes in protein expression related to iron metabolism in the kidneys of mice with UUO. (A) Representative immunoblots for transferrin receptor (TfR), divalent metal transporter-1 (DMT1), ferroportin (FPN), ferritin heavy chain (FTH), and ferritin light chain (FTL). (B) Quantitative densitometry analysis of TfR, DMT1, FPN, FTH, and FTL normalized to tubulin. Values are expressed as the mean ± SEM. *P<0.05, **P<0.01. *n* = 12 per group. (C) Representative immunohistochemical staining and localization of TfR, DMT1, FPN, FTH, and FTL.

In the kidneys of UUO+VEH mice, TfR expression decreased; meanwhile, DMT1 and FPN expression increased 2.1-fold and 1.5-fold, respectively. Treatment of UUO mice with DFO restored the expression of TfR to the level in sham+VEH mice and reduced FPN expression by half. DFO treatment itself increased TfR expression by 3.7-fold but did not change the expression of DMT1 and FPN in the kidneys of sham mice. FTH and FTL expression in the kidney decreased by half when sham mice were treated with DFO. FTH and FTL expression was approximately 1.5-fold higher in UUO mice than in sham mice. Ferritin levels in UUO mice treated with DFO were similar to those in sham mice (Fig. 5A and B). In immunohistochemical analysis, the iron transporters and ferritin were primarily expressed in the renal tubules (Fig. 5C).

## Discussion

In the present study, we showed that iron deprivation induced by DFO treatment suppressed renal interstitial fibrosis as well as the expression of collagen I, III, and IV in mice with UUO. Iron chelation also prevented macrophage infiltration and the induction of inflammatory cytokines. We also showed that DFO diminished renal oxidative stress by inhibiting p22^phox^ upregulation and TGF-β-Smad signaling activation in UUO mice. Thus, the protective effect of iron chelation in UUO-induced renal interstitial fibrosis involves the inhibition of oxidative stress, inflammation, and pro-fibrotic signaling.

Iron participates directly in tissue fibrosis, and iron reduction has been shown to suppress fibrotic changes in the liver. Patients with iron overload diseases, such as thalassemia, or patients who receive numerous blood transfusions often present with hepatic fibrosis. In these patients, the progression of liver fibrosis can be delayed or prevented by iron chelation therapy [30]–[32]. Similarly, iron reduction by phlebotomy or a low-iron diet suppresses hepatic fibrosis in patients with hepatitis C [33]–[35] and suppresses non-alcohol fatty liver disease [36]. However, these liver diseases are generally not thought to be iron-related.

The ameliorating effects of iron reduction on tissue fibrosis have been examined in various experimental and animal models. Ishizaka et al. showed that DFO treatment prevented cardiovascular fibrosis induced by AngII by inhibiting oxidative stress and macrophage infiltration [13], [14]. They also reported that iron chelation suppressed AngII-induced TGF-β1 upregulation in the kidney [19] and heart [37]. A low-iron diet attenuated the expression of TGF-β1 and the upregulation of collagen III in a CKD rat model [21]. Indeed, TGF-β1 is a well-known major pro-fibrotic stimulator of collagen and extracellular matrix production in a variety of cell types [38]. Smad is a downstream target of TGF-β1 signaling, and the TGF-β1-Smad pathway is critical in fibrosis development [39]. In the kidney, TGF-β1 expression increased with UUO [40], and treatment with a neutralizing antibody to TGF-β1 ameliorated UUO-induced renal interstitial fibrosis [41]. Disruption of Smad3 diminished UUO-induced renal tubulointerstitial fibrosis, suggesting that Smad3 plays a crucial role in the development of fibrosis in kidney diseases [42], [43]. In the present study, UUO induced TGF-β1 expression and Smad3 phosphorylation. The activation of TGF-β1-Smad3 signaling in UUO mice were inhibited by the iron chelator DFO, leading to the amelioration of UUO-induced renal fibrosis. Thus, the inhibition of TGF-β1-Smad3 signaling by iron reduction contributes to the prevention of UUO-induced renal interstitial fibrosis. In addition, activated fibroblasts (also known as myofibroblasts) that express αSMA are a major source of extracellular matrix production [44]. In this study, UUO-induced αSMA expression was markedly reduced by DFO treatment. Furthermore, fibroblasts were activated by various cytokines including TGF-β [45], and TGF-β signaling was inhibited by DFO. Therefore, DFO may inhibit fibrosis by inhibiting the TGF-β pathway activation in fibroblasts.

UUO induces macrophage infiltration in the renal tubulointerstitium. The macrophages produce inflammatory cytokines, resulting in a detrimental cycle of tubulointerstitial fibrotic change [46]. Iron reduction reduces macrophage infiltration and inflammatory cytokines in the aorta and heart of Dahl-salt sensitive rats [15], a CKD model induced by 5/6 nephrectomy [21], and in the adipose tissue of KKAy mice [16]. Consistent with these findings, DFO treatment mitigated macrophage infiltration and the production of inflammatory cytokines such as MCP-1 and IL-1β in UUO mice, which contributed to the preventive effect of iron reduction on inflammation.

The epithelial mesenchymal transition (EMT) contributes to the development of renal interstitial fibrosis induced by UUO [47]. Epithelial cells de-differentiate and lose their epithelial cell surface markers, and myofibroblasts differentiate into fibroblasts. The altered cells express mesenchymal marker proteins such as αSMA and fibronectin. Similar to its role in fibrosis, TGF-β1-Smad3 signaling is a key mediator for triggering EMT *in vivo* [42]. Therefore, we postulate that the reduction of UUO-induced renal αSMA and fibronectin expression by DFO suppresses renal fibrosis by inhibiting EMT via TGF-β-Smad signaling. However, several studies have shown that EMT does not result in the generation of interstitial myofibroblasts in fibrotic renal disease models, and therefore the role of EMT has remained controversial [48]. Therefore, further studies are necessary to confirm the role of EMT in renal interstitial fibrosis.

Oxidative stress is considered a contributing factor in the progression of CKD, including renal fibrosis [49]. UUO, a widely used experimental model of renal fibrosis, induces oxidative stress; oxidative stress plays a crucial role in the pathogenesis of UUO in the kidney [50]. Iron causes oxidative stress through its catalysis of the Fenton reaction, and iron-derived oxidative stress participates in various diseases. Indeed, iron reduction can ameliorate pathological states, not only in heredity hemochromatosis, but also in non-iron overload diseases. In addition to inducing oxidative stress through the Fenton reaction, iron affects NADPH oxidase, which produces superoxide. UUO increased the renal expression of NADPH oxidase subunits such as p22^phox^, p47^phox^, and p67^phox^ [51]. Our study group and others have demonstrated that iron chelators suppress oxidative stress by inhibiting p22^phox^ expression and NADPH oxidase activity in diabetic obese mice [16], mice with diabetic nephropathy [23], a murine model with local inflammation [52], and in endothelial cells [53]. Consistent with these findings, iron deprivation by DFO suppressed the increase in NADPH oxidase activity and p22^phox^ expression in a UUO mouse model, thus providing insight into the mechanism by which iron reduction affects renal fibrosis through decreased oxidative stress. Sugiyama et al. showed that UUO-induced p22^phox^ was expressed in renal tubules [51]. We previously showed that dietary iron restriction reduced the upregulation of renal p22^phox^ expression in renal proximal tubules of *db/db* mice [23]. Furthermore, p22^phox^ expression was colocalized in F4/80- or CD 68-positive cells that were obtained from the fat of diabetic and obese mice or from human atherosclerotic coronary arteries [16], [54]. Therefore, the reduction of UUO-induced renal macrophage infiltration after DFO treatment may have further decreased NADPH oxidase activity. However, various cell types, such as fibroblasts, endothelial cells, podocytes, and pericytes/perivascular fibroblasts, produce NADPH oxidase, which is involved in the progression of renal fibrosis. Further studies are required to elucidate whether DFO affects NADPH oxidase production in various cell types.

The kidney is thought to participate in iron homeostasis because renal tubular cells express iron importers, TfR [55] and DMT1 [56], [57], and an iron exporter, FPN [58]. Expression of these iron transporters was altered in diabetic nephropathy [23], [59], 5/6 nephrectomy [21], the anemic kidney [60], and kidneys with AngII infusion [20], indicating an alteration in renal iron metabolism in these diseases. We observed that iron concentration was increased in kidney of UUO mice with reduced TfR expression and increased DMT1 and FPN expression in the renal proximal tubular cells. In this study, the alterations in iron transporter expression may be compensatory changes that prevent renal iron elevation induced by UUO. Additionally, the expression of heavy and light chain ferritin was also increased in the renal tubules of UUO mice. In other animal models, ferritin expression increased in the aorta and heart after AngII infusion [13], [14], the aorta and heart of Dahl salt-sensitive rats [15], the adipose tissue of KKAy mice [16], and the kidneys of *db/db* mice with diabetic nephropathy [23]. Ferritin, an intracellular iron storage protein, reduces intracellular free iron levels, thereby suppressing catalysis of the Fenton reaction [61]. Indeed, tissue iron concentration are also augmented in the aorta and heart after AngII infusion [13], [14], aorta of Dahl salt-sensitive rats [15], and the kidneys of *db/db* mice [23]. Accordingly to those, whole kidney iron concentration and renal ferritin levels were elevated in UUO mice, and upregulated ferritin levels may be a compensatory manner as a result of increased free iron levels, thus contributing to reduced oxidative stress via the Fenton reaction.

Iron chelation by DFO has protective effects in various disease models, including renal fibrosis. However, several studies have shown adverse effects of DFO on kidney injury. DFO promotes HIF-1α activity [25], and HIF-1α activation exacerbates UUO-induced renal fibrosis via the EMT [26]. In a rat model of ischemia/reperfusion renal injury, IL-10 infusion protected against renal injury by inducing lipocalin-2 in an intracellular iron-dependent manner; the effect of IL-10 were abolished by DFO treatment [28]. Thus, the effect of DFO on renal injury is controversial and unresolved. Further studies are necessary to determine whether iron chelation therapy alleviates or aggravates tubulointerstitial fibrosis.

**Figure 6:**
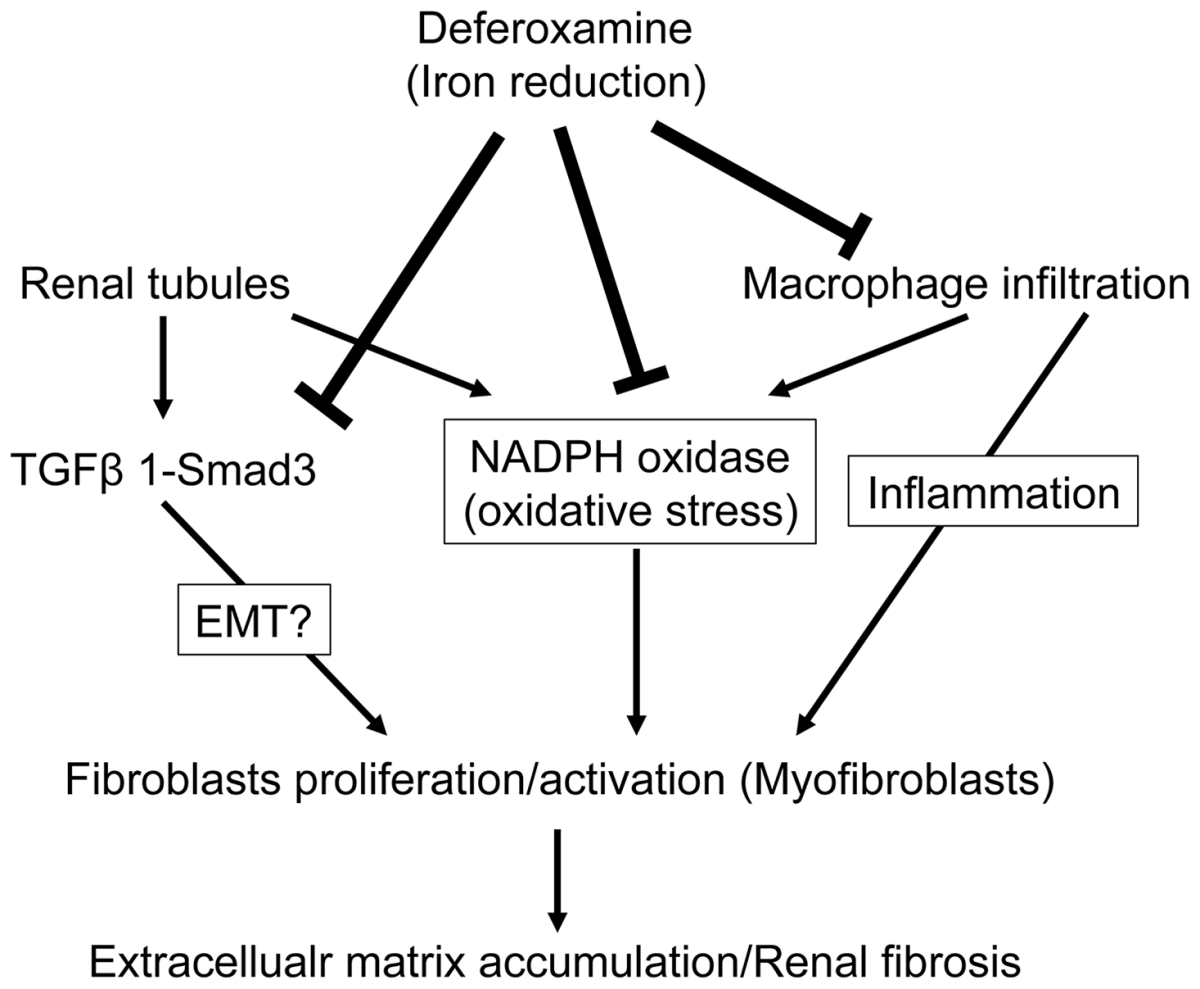
The schema of the proposed mechanism for the inhibitory effects of DFO on UUO-induced renal interstitial fibrosis.

In conclusion, iron reduction by DFO prevents renal tubulointerstitial fibrosis by inhibiting oxidative stress and TGF-β1-Smad3 signaling activation. The hypothetical mechanism by which DFO affects UUO-induced renal interstitial fibrosis is shown in Figure 6. Iron reduction is a promising therapeutic strategy for CKD.

## References

[1] Collins AJ, Foley RN, Gilbertson DT, Chen SC (2009) The state of chronic kidney disease, ESRD, and morbidity and mortality in the first year of dialysis. Clin J Am Soc Nephrol 4 Suppl 1S5–11. 19996006 10.2215/CJN.05980809

[2] Go AS, Chertow GM, Fan D, McCulloch CE, Hsu CY (2004) Chronic kidney disease and the risks of death, cardiovascular events, and hospitalization. N Engl J Med 351: 1296–1305. 15385656 10.1056/NEJMoa041031

[3] Iwano M, Neilson EG (2004) Mechanisms of tubulointerstitial fibrosis. Curr Opin Nephrol Hypertens 13: 279–284. 15073485 10.1097/00041552-200405000-00003

[4] Kruszewski M (2003) Labile iron pool: the main determinant of cellular response to oxidative stress. Mutat Res 531: 81–92. 14637247 10.1016/j.mrfmmm.2003.08.004

[5] Cairo G, Recalcati S, Pietrangelo A, Minotti G (2002) The iron regulatory proteins: targets and modulators of free radical reactions and oxidative damage. Free Radic Biol Med 32: 1237–1243. 12057761 10.1016/s0891-5849(02)00825-0

[6] Camaschella C (2005) Understanding iron homeostasis through genetic analysis of hemochromatosis and related disorders. Blood 106: 3710–3717. 16030190 10.1182/blood-2005-05-1857

[7] Hayashi H, Takikawa T, Nishimura N, Yano M, Isomura T, et al (1994) Improvement of serum aminotransferase levels after phlebotomy in patients with chronic active hepatitis C and excess hepatic iron. Am J Gastroenterol 89: 986–988. 8017395

[8] Hayashi H, Takikawa T, Nishimura N, Yano M (1995) Serum aminotransferase levels as an indicator of the effectiveness of venesection for chronic hepatitis C. J Hepatol. 22: 268–271. 10.1016/0168-8278(95)80278-9 7608476

[9] Smith MA, Harris PL, Sayre LM, Perry G (1997) Iron accumulation in Alzheimer disease is a source of redox-generated free radicals. Proc Natl Acad Sci U S A 94: 9866–9868. 9275217 10.1073/pnas.94.18.9866 PMC23283

[10] Liu B, Moloney A, Meehan S, Morris K, Thomas SE, et al (2011) Iron promotes the toxicity of amyloid beta peptide by impeding its ordered aggregation. J Biol Chem 286: 4248–4256. 21147772 10.1074/jbc.M110.158980 PMC3039358

[11] Kimura F, Hayashi H, Yano M, Yoshioka K, Matsumura T, et al (2005) Additional effect of low iron diet on iron reduction therapy by phlebotomy for chronic hepatitis C. Hepatogastroenterology. 52: 563–566. 15816478

[12] Cuajungco MP, Faget KY, Huang X, Tanzi RE, Bush AI (2000) Metal chelation as a potential therapy for Alzheimer’s disease. Ann N Y Acad Sci 920: 292–304. 11193167 10.1111/j.1749-6632.2000.tb06938.x

[13] Ishizaka N, Saito K, Mitani H, Yamazaki I, Sata M, et al (2002) Iron overload augments angiotensin II-induced cardiac fibrosis and promotes neointima formation. Circulation 106: 1840–1846. 12356639 10.1161/01.cir.0000031161.77536.02

[14] Ishizaka N, Saito K, Mori I, Matsuzaki G, Ohno M, et al (2005) Iron chelation suppresses ferritin upregulation and attenuates vascular dysfunction in the aorta of angiotensin II-infused rats. Arterioscler Thromb Vasc Biol 25: 2282–2288. 16100038 10.1161/01.ATV.0000181763.57495.2b

[15] Naito Y, Hirotani S, Sawada H, Akahori H, Tsujino T, et al (2011) Dietary iron restriction prevents hypertensive cardiovascular remodeling in Dahl salt-sensitive rats. Hypertension 57: 497–504. 21263124 10.1161/HYPERTENSIONAHA.110.159681

[16] Tajima S, Ikeda Y, Sawada K, Yamano N, Horinouchi Y, et al (2012) Iron reduction by deferoxamine leads to amelioration of adiposity via the regulation of oxidative stress and inflammation in obese and type 2 diabetes KKAy mice. Am J Physiol Endocrinol Metab 302: E77–86. 21917632 10.1152/ajpendo.00033.2011

[17] Cooksey RC, Jones D, Gabrielsen S, Huang J, Simcox JA, et al (2010) Dietary iron restriction or iron chelation protects from diabetes and loss of beta-cell function in the obese (ob/ob lep−/−) mouse. Am J Physiol Endocrinol Metab 298: E1236–1243. 20354157 10.1152/ajpendo.00022.2010 PMC2886527

[18] Minamiyama Y, Takemura S, Kodai S, Shinkawa H, Tsukioka T, et al (2010) Iron restriction improves type 2 diabetes mellitus in Otsuka Long-Evans Tokushima fatty rats. Am J Physiol Endocrinol Metab 298: E1140–1149. 20215574 10.1152/ajpendo.00620.2009

[19] Saito K, Ishizaka N, Aizawa T, Sata M, Iso ON, et al (2004) Role of aberrant iron homeostasis in the upregulation of transforming growth factor-beta1 in the kidney of angiotensin II-induced hypertensive rats. Hypertens Res 27: 599–607. 15492480 10.1291/hypres.27.599

[20] Ishizaka N, Saito K, Furuta K, Matsuzaki G, Koike K, et al (2007) Angiotensin II-induced regulation of the expression and localization of iron metabolism-related genes in the rat kidney. Hypertens Res 30: 195–202. 17460390 10.1291/hypres.30.195

[21] Naito Y, Fujii A, Sawada H, Hirotani S, Iwasaku T, et al (2012) Effect of iron restriction on renal damage and mineralocorticoid receptor signaling in a rat model of chronic kidney disease. J Hypertens 30: 2192–2201. 22922699 10.1097/HJH.0b013e3283581a64

[22] Naito Y, Fujii A, Sawada H, Hirotani S, Iwasaku T, et al (2013) Dietary iron restriction prevents further deterioration of renal damage in a chronic kidney disease rat model. J Hypertens 31: 1203–1213. 23552122 10.1097/HJH.0b013e328360381d

[23] Ikeda Y, Enomoto H, Tajima S, Izawa-Ishizawa Y, Kihira Y, et al (2013) Dietary iron restriction inhibits progression of diabetic nephropathy in db/db mice. Am J Physiol Renal Physiol 304: F1028–1036. 23389454 10.1152/ajprenal.00473.2012

[24] Neufeld EJ (2006) Oral chelators deferasirox and deferiprone for transfusional iron overload in thalassemia major: new data, new questions. Blood 107: 3436–3441. 16627763 10.1182/blood-2006-02-002394 PMC1895765

[25] Wang GL, Semenza GL (1993) Desferrioxamine induces erythropoietin gene expression and hypoxia-inducible factor 1 DNA-binding activity: implications for models of hypoxia signal transduction. Blood 82: 3610–3615. 8260699

[26] Higgins DF, Kimura K, Bernhardt WM, Shrimanker N, Akai Y, et al (2007) Hypoxia promotes fibrogenesis in vivo via HIF-1 stimulation of epithelial-to-mesenchymal transition. J Clin Invest 117: 3810–3820. 18037992 10.1172/JCI30487 PMC2082142

[27] Norman JT, Clark IM, Garcia PL (2000) Hypoxia promotes fibrogenesis in human renal fibroblasts. Kidney Int 58: 2351–2366. 11115069 10.1046/j.1523-1755.2000.00419.x

[28] Jung M, Sola A, Hughes J, Kluth DC, Vinuesa E, et al (2012) Infusion of IL-10-expressing cells protects against renal ischemia through induction of lipocalin-2. Kidney Int 81: 969–982. 22278021 10.1038/ki.2011.446

[29] Ikeda Y, Tajima S, Izawa-Ishizawa Y, Kihira Y, Ishizawa K, et al (2012) Estrogen Regulates Hepcidin Expression via GPR30-BMP6-Dependent Signaling in Hepatocytes. PLoS One 7: e40465. 22792339 10.1371/journal.pone.0040465 PMC3394730

[30] Barry M, Flynn DM, Letsky EA, Risdon RA (1974) Long-term chelation therapy in thalassaemia major: effect on liver iron concentration, liver histology, and clinical progress. Br Med J 2: 16–20. 4821036 10.1136/bmj.2.5909.16 PMC1610120

[31] Olivieri NF, Brittenham GM, McLaren CE, Templeton DM, Cameron RG, et al (1998) Long-term safety and effectiveness of iron-chelation therapy with deferiprone for thalassemia major. N Engl J Med 339: 417–423. 9700174 10.1056/NEJM199808133390701

[32] Harmatz P, Butensky E, Quirolo K, Williams R, Ferrell L, et al (2000) Severity of iron overload in patients with sickle cell disease receiving chronic red blood cell transfusion therapy. Blood 96: 76–79. 10891433

[33] Yano M, Hayashi H, Wakusawa S, Sanae F, Takikawa T, et al (2002) Long term effects of phlebotomy on biochemical and histological parameters of chronic hepatitis C. Am J Gastroenterol. 97: 133–137. 10.1111/j.1572-0241.2002.05436.x 11808937

[34] Sartori M, Andorno S, Rigamonti C, Boldorini R (2001) Chronic hepatitis C treated with phlebotomy alone: biochemical and histological outcome. Dig Liver Dis 33: 157–162. 11346145 10.1016/s1590-8658(01)80072-4

[35] Kato J, Kobune M, Nakamura T, Kuroiwa G, Takada K, et al (2001) Normalization of elevated hepatic 8-hydroxy-2′-deoxyguanosine levels in chronic hepatitis C patients by phlebotomy and low iron diet. Cancer Res 61: 8697–8702. 11751387

[36] Facchini FS, Hua NW, Stoohs RA (2002) Effect of iron depletion in carbohydrate-intolerant patients with clinical evidence of nonalcoholic fatty liver disease. Gastroenterology 122: 931–939. 11910345 10.1053/gast.2002.32403

[37] Saito K, Ishizaka N, Aizawa T, Sata M, Iso-o N, et al (2005) Iron chelation and a free radical scavenger suppress angiotensin II-induced upregulation of TGF-beta1 in the heart. Am J Physiol Heart Circ Physiol 288: H1836–1843. 15550525 10.1152/ajpheart.00679.2004

[38] Massague J (1990) The transforming growth factor-beta family. Annu Rev Cell Biol 6: 597–641. 2177343 10.1146/annurev.cb.06.110190.003121

[39] Massague J (2000) How cells read TGF-beta signals. Nat Rev Mol Cell Biol 1: 169–178. 11252892 10.1038/35043051

[40] Kaneto H, Morrissey J, Klahr S (1993) Increased expression of TGF-beta 1 mRNA in the obstructed kidney of rats with unilateral ureteral ligation. Kidney Int 44: 313–321. 8377375 10.1038/ki.1993.246

[41] Miyajima A, Chen J, Lawrence C, Ledbetter S, Soslow RA, et al (2000) Antibody to transforming growth factor-beta ameliorates tubular apoptosis in unilateral ureteral obstruction. Kidney Int 58: 2301–2313. 11115064 10.1046/j.1523-1755.2000.00414.x

[42] Sato M, Muragaki Y, Saika S, Roberts AB, Ooshima A (2003) Targeted disruption of TGF-beta1/Smad3 signaling protects against renal tubulointerstitial fibrosis induced by unilateral ureteral obstruction. J Clin Invest 112: 1486–1494. 14617750 10.1172/JCI19270 PMC259132

[43] Inazaki K, Kanamaru Y, Kojima Y, Sueyoshi N, Okumura K, et al (2004) Smad3 deficiency attenuates renal fibrosis, inflammation,and apoptosis after unilateral ureteral obstruction. Kidney Int 66: 597–604. 15253712 10.1111/j.1523-1755.2004.00779.x

[44] Tomasek JJ, Gabbiani G, Hinz B, Chaponnier C, Brown RA (2002) Myofibroblasts and mechano-regulation of connective tissue remodelling. Nat Rev Mol Cell Biol 3: 349–363. 11988769 10.1038/nrm809

[45] Alvarez RJ, Sun MJ, Haverty TP, Iozzo RV, Myers JC, et al (1992) Biosynthetic and proliferative characteristics of tubulointerstitial fibroblasts probed with paracrine cytokines. Kidney Int 41: 14–23. 1593850 10.1038/ki.1992.3

[46] Vielhauer V, Anders HJ, Mack M, Cihak J, Strutz F, et al (2001) Obstructive nephropathy in the mouse: progressive fibrosis correlates with tubulointerstitial chemokine expression and accumulation of CC chemokine receptor 2- and 5-positive leukocytes. J Am Soc Nephrol 12: 1173–1187. 11373340 10.1681/ASN.V1261173

[47] Grande MT, Lopez-Novoa JM (2009) Fibroblast activation and myofibroblast generation in obstructive nephropathy. Nat Rev Nephrol 5: 319–328. 19474827 10.1038/nrneph.2009.74

[48] Grgic I, Duffield JS, Humphreys BD (2012) The origin of interstitial myofibroblasts in chronic kidney disease. Pediatr Nephrol 27: 183–193. 21311912 10.1007/s00467-011-1772-6 PMC3116994

[49] Oberg BP, McMenamin E, Lucas FL, McMonagle E, Morrow J, et al (2004) Increased prevalence of oxidant stress and inflammation in patients with moderate to severe chronic kidney disease. Kidney Int 65: 1009–1016. 14871421 10.1111/j.1523-1755.2004.00465.x

[50] Kawada N, Moriyama T, Ando A, Fukunaga M, Miyata T, et al (1999) Increased oxidative stress in mouse kidneys with unilateral ureteral obstruction. Kidney Int 56: 1004–1013. 10469368 10.1046/j.1523-1755.1999.00612.x

[51] Sugiyama H, Kobayashi M, Wang DH, Sunami R, Maeshima Y, et al (2005) Telmisartan inhibits both oxidative stress and renal fibrosis after unilateral ureteral obstruction in acatalasemic mice. Nephrol Dial Transplant 20: 2670–2680. 16141465 10.1093/ndt/gfi045

[52] Li L, Frei B (2006) Iron chelation inhibits NF-kappaB-mediated adhesion molecule expression by inhibiting p22(phox) protein expression and NADPH oxidase activity. Arterioscler Thromb Vasc Biol 26: 2638–2643. 16973969 10.1161/01.ATV.0000245820.34238.da

[53] Li L, Frei B (2009) Prolonged exposure to LPS increases iron, heme, and p22phox levels and NADPH oxidase activity in human aortic endothelial cells: inhibition by desferrioxamine. Arterioscler Thromb Vasc Biol 29: 732–738. 19251588 10.1161/ATVBAHA.108.183210 PMC2724965

[54] Azumi H, Inoue N, Takeshita S, Rikitake Y, Kawashima S, et al (1999) Expression of NADH/NADPH oxidase p22phox in human coronary arteries. Circulation 100: 1494–1498. 10510050 10.1161/01.cir.100.14.1494

[55] Fuller SD, Simons K (1986) Transferrin receptor polarity and recycling accuracy in “tight” and “leaky” strains of Madin-Darby canine kidney cells. J Cell Biol 103: 1767–1779. 2877994 10.1083/jcb.103.5.1767 PMC2114390

[56] Ferguson CJ, Wareing M, Ward DT, Green R, Smith CP, et al (2001) Cellular localization of divalent metal transporter DMT-1 in rat kidney. Am J Physiol Renal Physiol 280: F803–814. 11292622 10.1152/ajprenal.2001.280.5.F803

[57] Canonne-Hergaux F, Gros P (2002) Expression of the iron transporter DMT1 in kidney from normal and anemic mk mice. Kidney Int 62: 147–156. 12081573 10.1046/j.1523-1755.2002.00405.x

[58] Wolff NA, Liu W, Fenton RA, Lee WK, Thevenod F, et al (2011) Ferroportin 1 is expressed basolaterally in rat kidney proximal tubule cells and iron excess increases its membrane trafficking. J Cell Mol Med 15: 209–219. 20015204 10.1111/j.1582-4934.2009.00985.x PMC3822789

[59] Ward DT, Hamilton K, Burnand R, Smith CP, Tomlinson DR, et al (2005) Altered expression of iron transport proteins in streptozotocin-induced diabetic rat kidney. Biochim Biophys Acta 1740: 79–84. 15878745 10.1016/j.bbadis.2005.01.008

[60] Ferguson CJ, Wareing M, Delannoy M, Fenton R, McLarnon SJ, et al (2003) Iron handling and gene expression of the divalent metal transporter, DMT1, in the kidney of the anemic Belgrade (b) rat. Kidney Int 64: 1755–1764. 14531808 10.1046/j.1523-1755.2003.00274.x

[61] Arosio P, Ingrassia R, Cavadini P (2009) Ferritins: a family of molecules for iron storage, antioxidation and more. Biochim Biophys Acta 1790: 589–599. 18929623 10.1016/j.bbagen.2008.09.004

